# The winter, the summer and the summer dream of artificial intelligence in law: Presidential address to the 18th International Conference on Artificial Intelligence and Law

**DOI:** 10.1007/s10506-022-09309-8

**Published:** 2022-02-03

**Authors:** Enrico Francesconi

**Affiliations:** 1 Consiglio Nazionale delle Ricerche, Istituto di Informatica Giuridica e Sistemi Giudiziari (IGSG-CNR), Firenze, Italy; 2 European Parliament, Luxembourg, Luxembourg

**Keywords:** AI, Semantic web, Provisions, Norms, Smart data, Gödel theorem

## Abstract

This paper reflects my address as IAAIL president at ICAIL 2021. It is aimed to give my vision of the status of the AI and Law discipline, and possible future perspectives. In this respect, I go through different seasons of AI research (of AI and Law in particular): from the Winter of AI, namely a period of mistrust in AI (throughout the eighties until early nineties), to the Summer of AI, namely the current period of great interest in the discipline with lots of expectations. One of the results of the first decades of AI research is that “intelligence requires knowledge”. Since its inception the Web proved to be an extraordinary vehicle for knowledge creation and sharing, therefore it’s not a surprise if the evolution of AI has followed the evolution of the Web. I argue that a bottom-up approach, in terms of machine/deep learning and NLP to extract knowledge from raw data, combined with a top-down approach, in terms of legal knowledge representation and models for legal reasoning and argumentation, may represent a promotion for the development of the Semantic Web, as well as of AI systems. Finally, I provide my insight in the potential of AI development, which takes into account technological opportunities and theoretical limits.

## Introduction

I’m very glad and honoured to give the presidential address at the ICAIL 2021. First of all let me say that it’s been a pleasure to serve the IAAIL community as President for the period 2020-2021, and with this talk I’m pleased to give an overview of the journey of the ICAIL conference series from my perspective, throughout different seasons of AI research and the possible developments.

I’m part of a generation that joined IAAIL^1^ and ICAIL^2^ in the early 2000s, when the pioneers in AI and Law had already established the foundations of this discipline and a new generation of researchers joined as well, during a phase characterized by a specific level of maturity of the Web revolution. In such a revolution I like to spot a singularity for the AI and Law domain represented by the Semantic Web development.

There is no doubt, in fact, that keywords like Semantic Web, Web 3.0, Linked Open Data, Smart Data, Ontology, as well as Machine Learning, Natural Language Processing and, eventually, Artificial Intelligence are concepts of interest not only in the Information Technology field, but also identify a specific research area for the Law.

In this very field, the first who identified a close relationship between Computer Science and Law was Lee Loevinger, judge of the Supreme Court of Minnesota, who used the term *jurimetrics* to indicate a way to approach the Law inspired by computational methods (Loevinger 1949). Among the pioneers, I like in particular to recall the National Research Council of Italy^3^, that organised a series of conferences on “Logica, Informatica e Diritto”^4^ throughout the ’80s: the first in 1981, than in 1985, finally in 1989. In the same period (late ’80s) the International Association for AI and Law (IAAIL) was established with the aim to support, develop and promote the field of AI and Law at the international level.

## The AI winter

**Fig. 1:**
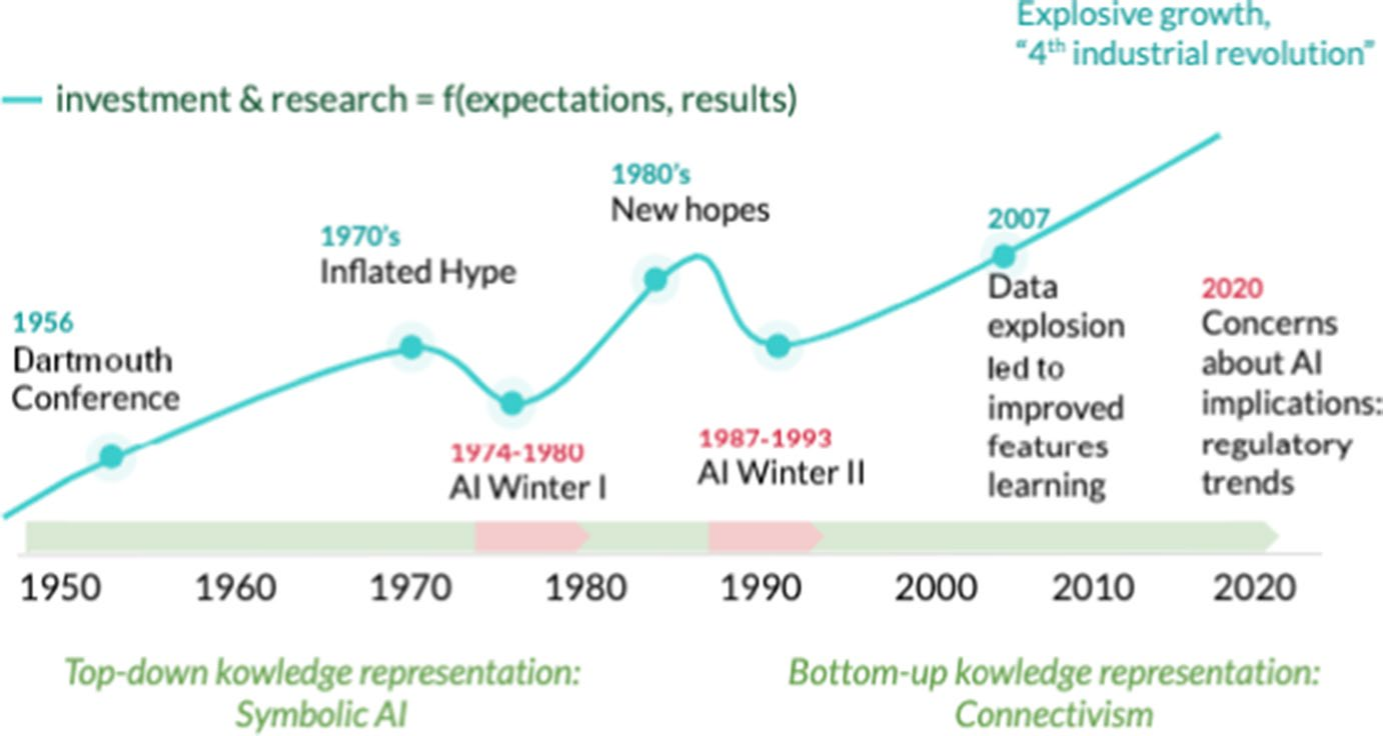
The AI evolution over time

AI and Law research pioneers developed their first studies (throughout the ’80s) in the so-called *AI-Winter* (Fig. 1), namely a climate of generalized mistrust towards the possible developments of AI.

Many have been the reasons for such a mistrust: theoretical studies had led to experimental applications of limited scope, difficult to scale in real scenarios due to the cost and complexity of representing and maintaining the necessary amount of information. Furthermore, it was immediately clear that not all information can be represented in symbolic form (for example visual information). Moreover, the attempts to manage sub-symbolic information, as in the case of the first connectionist models, clashed with the limits of such computational structures, as for example in the Rosenblatt’s perceptron (Rosenblatt 1958) with the famous XOR problem pitfalls (Minsky and Papert 1969). For these reasons, more specific terminologies were preferred to the term Artificial Intelligence: they were aimed to indicate particular sectors of deductive and interpretative automation processes, inspired by some functions of human intelligence (Sartor 2016). We rather spoke of Information Retrieval, Pattern Recognition, Expert Systems, Probabilistic Reasoning, etc., terms which are probably less evocative but they had the purpose of creating more limited expectations for certain application fields.

## The end of AI and Law winter

Nowadays, Artificial Intelligence is surrounded by a lot of hype, and this holds for the AI and Law domain too, testified to also by the relevant number of participants to ICAIL 2021, organized on-line because of the Covid-19 pandemic. It was a hard decision to go for the virtual version of the conference, but our Brazilian colleagues of the University of Saõ Paulo have taken the risk, refocusing on the opportunity of the on-line version. Finally, the challenge was won, as demonstrated by the participation figures: about 500 participants in the pre-event, 1380 in the main conference, 11 workshops, 89 paper submissions.

This success comes from afar and it is the result of a long journey, during which, according to Thorne McCarty^5^, the research on AI and Law made a lot of progress along two distinct direction lines and motivations: theoretical and practical (McCarty 1990). On the theoretical side, the aim is to gain a better understanding of the process of legal reasoning and legal argumentation, using computational models and techniques. On the practical side, the aim is to build intelligent legal information systems supporting legal practitioners, decision makers and citizens (McCarty 1990).

In this respect McCarty underlined key questions which, in the last few years, have characterised the research in AI and Law: How much of legal reasoning can be reduced to reasoning with rules? How is it possible to reason with cases at all? Is it possible to develop a computational theory of legal arguments? (McCarty 1990)

According to McCarty, a possible solution to such questions is the development of systems based on “deep conceptual models” of the relevant domain (McCarty 1984). He identified the main obstacle to computational models for legal reasoning in the knowledge representation problem (McCarty 1990). That’s why at ICAIL 1989 he published a paper about “A Language for Legal Discourse”, underlining the need of a language able to represent concepts, states, events, actions, and all of the deontic modalities.

On the other side, Trevor Bench Capon argued that for most practical applications, intelligent information systems can be built without “deep conceptual models” (Bench-Capon 1989). According to Bench Capon an expert system can be based on a formalisation of the legislation itself, and a faithful representation of the legal rules (Bench-Capon 1989), this way introducing the key concept of *Isomorphism*, which inspired much AI and Law research in the following years. Twenty years later Trevor Bench Capon and Tom Gordon’s paper “Isomorphism and Argumentation” (Bench-Capon and Gordon 2009) brought this very analysis up to date. Moreover, Bench Capon in 1993 wrote an interesting paper aimed to complement the concept of isomorphism, showing an exercise aimed to extract the rationale underlying legal decisions given only a set of decided cases (Bench-Capon 1993).

At first sight these seem two opposite positions, but I conceived them as complementary, as they address two view points which would characterise the research in AI and Law in the following decades, developed in terms of rule-based or case-based reasoning, as well as, in other terms, knowledge systems or data systems, respectively, laying the foundations for the end of AI Winter in the AI and Law domain.

In fact, in the succeeding years, a relevant number of works have been carried out concerning legal reasoning based on “open-textured” concepts, non-monotonic/defeasible reasoning (Gordon 1988, 1987; Antoniou et al. 2008), rule-based approaches to defeasible reasoning (Gardner 1987), case-based legal reasoning (Aleven and Ashley 1997), preferences over rules in non-monotonic reasoning, models for adversarial legal reasoning (Prakken and Sartor 1997, 1996), deontic logic (Sartor 2006; Francesconi 2016). Other works addressed the theory of legal argumentation Walton (2006), dialogues between parties, analysis of rules and precedents, persuasion and values in legal arguments, argumentation schemes Atkinson et al. (2017), as well as arguments and stories Bex (2015). More recently the aspects of ethics and explainable AI have also gained lots of interest Atkinson et al. (2020) Barredo Arrieta et al. (2020).

On the other hand, in the last few years the area of data systems has been highly developed [23] (Conrad and Zeleznikow 2015), in parallel with the success of the application of connectionist models to the legal domain Bochereau et al. (1991), in fields like legal information retrieval and eDiscovery (Conrad 2010), semantic annotation of legal texts (Biagioli et al. 2005) (Francesconi and Passerini 2007), arguments extraction (Mochales Palau and Moens 2009), legal predictions (Savelka et al. 2021), legal text summarization (Bhattacharya et al. 2021), legal network analysis (Winkels and J.d.R. 2011), quantitative reasoning (Lauristen 2015). In the very sense of data systems, the affirmation of emerging deep learning methodologies (Mikolov et al. 2013; Devlin et al. 2019) is representing a well established reality which opens up to new frontiers in machine learning and AI services for the legal domain.

The first attempts to bridge the gap between “case-based” and “rule-based” systems were carried out by the works of Kevin Ashley and Edwina Rissland. In such works, they addressed the problem of reasoning with cases and hypotheticals (Rissland and Ashley 1987; Ashley 1991), using elementary logic as well as notions of relevant similarities and differences between cases and analogous precedents. In particular, they underlined the roles of precedents in legal arguments and hypotheticals, as well as they combined rules and cases to solve case-based reasoning problems (Rissland and Skalak 1989, 1991). A similar hybrid approach was followed by Karl Branting, who aimed to combine rules and structured cases to determine and justify the legal consequences of a given set of facts (Branting 1991).

The combination of knowledge systems and data systems and, more specifically for the legal domain, the unification of rule-based and case-based systems, has been recently theorized by Bart Verheij, in his presidential address at ICAIL 2019, in terms of argumentation systems, where the focus is on hybrid critical discussion “where different hypothetical perspectives are constructed and evaluated until a good answer is found”. He spoke of “AI as Law” in the sense of the development of hybrid critical discussion systems (Verheij 2020).

## In the summer of AI and law

The debate and the relevant number of works developed within the AI community, as well as within the AI and Law community as previously addressed, took us out of the AI Winter period, leading us to a new era of AI and Law, characterized by concrete expectations and new awareness.

From the analysis of the literature in AI and Law we can conclude that, while lots of theoretical outcomes have been achieved in terms of both symbolic and sub-symbolic (or connectionist) AI, a limited number of large scale applications still exist in this field. It is therefore reasonable to wonder why this happened. My opinion is that this phenomenon is due to the strict relations and dependencies between *AI* and *Knowledge*.

According to Elaine Rich and Kevin Knight, one of the results of the first decades of AI research is that “intelligence requires knowledge” (Rich and Knight 1991). To compensate its indispensability, *Knowledge* has less desirable properties: it’s voluminous, it’s hard to characterise accurately, it is constantly changing, it differs from data because it needs a semantic organization (Rich and Knight 1991).

In my opinion, one of the main reasons of the AI Winter was the insufficient amount of *Knowledge* available. But in the early ’90s AI meets the Web and this changes everything. Since its inception, the Web proved to be an extraordinary vehicle for knowledge creation and sharing. Thanks to the availability of large quantities of information in digital format, the Web appeared as a crucial component for the creation of AI systems. On the other hand, the Internet and the Web require advanced AI applications for managing and selecting information. For these reasons, in addition to the natural evolution of algorithms and technology, it was the very meeting of AI with the Web that determined the end to the AI Winter, giving new impetus to the study of AI systems.

In this scenario the evolution of AI has followed the evolution of the Web. Of particular importance is the age of the Web known as *Semantic Web* (or Web 3.0), namely an environment of semantic interoperability of data, objects and agents. The Semantic Web represents an environment of adaptive knowledge consisting of “Smart Data”, essential for the development of AI systems. Knowledge, in fact, is nothing but data and semantics, therefore Knowledge *is in* the Semantic Web, which represents an essential infrastructure for AI systems.

Knowledge available in the Semantic Web is essential for the AI and Law domain, too. In fact, the Semantic Web provides knowledge models for a top-down approach to AI and Law, in terms of legal knowledge representation, models for legal reasoning and argumentation, planning and explainability. Moreover, the Semantic Web provides data, in particular *Smart Data*, for a bottom-up approach to the AI and Law, in terms of machine/deep learning and NLP approach for rule-based or case-based systems, argument mining, legal information discovery and retrieval (Francesconi and Passerini 2007; de Maat et al. 2010; Peters and Wyner 2016; Lenci et al. 2007; B. Waltl et al. 2017).

Nowadays we are clearly in the Summer of AI, in particular for AI and Law, with lots of opportunities to develop intelligent systems. One of the reasons of the explosion of such a season is the maturity of the Semantic Web as infrastructure for AI: in fact, the Semantic Web provides languages for knowledge representation systems, as well as Smart Data for intelligent applications. Moreover, from the early debate in the AI and Law community, it was clear that for developing intelligent legal information systems we need knowledge models and languages for legal rules description, algorithms within specific logic profiles for the activation of such rules (legal reasoning), as well as Smart Data. This is actually what the Semantic Web does: it represents a knowledge infrastructure for AI and Law, it provides standards and languages for legal knowledge representation, Smart Data for legal autonomous agents to mimic intelligent behaviour, as well as being a stimulus for machine learning approaches able to represent the Law as code. This is the precondition to develop AI applications in large scale for the Law domain.

In this context, a relevant number of works has been developed (Casanovas et al. 2016; Boer et al. 2010). In particular Kevin Ashely pointed out the importance of ontologies for analogical legal arguments (Ashley 2011), in order to support case-based comparisons between problems and cases, to distinguish deep and shallow analogies, as well as to induce/test hypotheses (hypothetical reasoning).

## The scenario of my research

This is also the scenario of my research, aimed at implementing AI solutions in the legal domain by representing legal rules as code, namely rules amenable for computation in the Semantic Web. The most part of my work has been addressed to represent legal rules at two levels of abstraction: in terms of a set of signs organized in words and sentences for creating normative statements, typically called *Provisions* (Raz 1980; Biagioli 2009), as well as in terms of the meaning for application of such normative statements, typically called *Norms* (Guastini 2010; Marmor 2014).

**Fig. 2:**
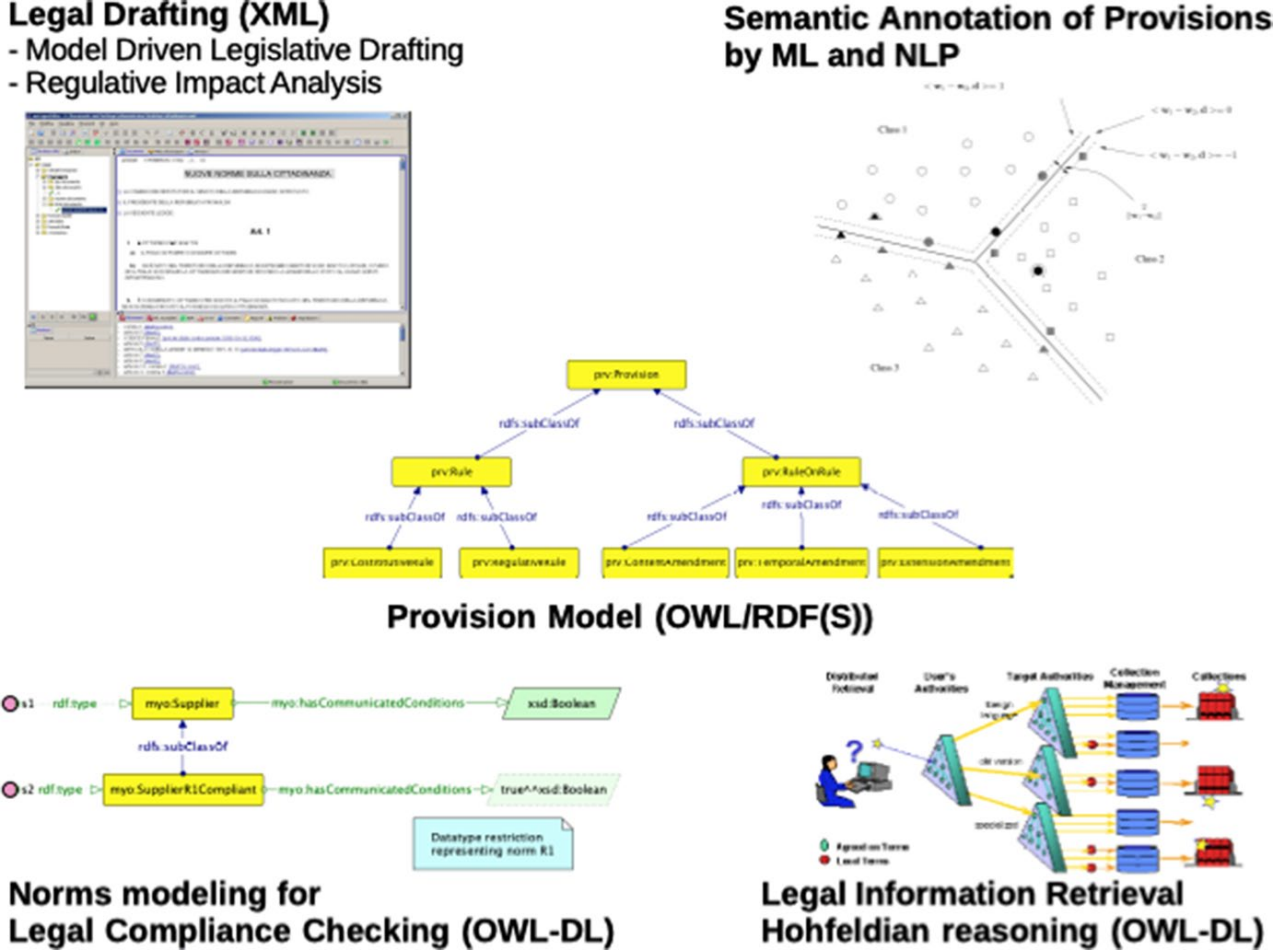
The topics of my research

Provisions and norms have, therefore, different roles and properties pertaining to different abstraction levels. A provision, as pure textual object, represents the building block of the legal order (provisions can enter, leave or modify the legal order itself). On the other hand, a norm represents the applicative interpretation of a legal rule in a real scenario, and it can introduce restrictions on the real world (in case of obligations, for example). Using Semantic Web technologies (Fig. 2) provisions are modeled by the Provision Model, which can be used for implementing model driven legislative drafting solutions (Biagioli et al. 2005a, 2007; Agnoloni et al. 2007), for semantic annotation of legal texts, in case supported by machine learning and natural language processing facilities (Biagioli et al. 2005; Francesconi and Passerini 2007), for advanced legal information retrieval and reasoning (as Hohfeldian reasoning) (Francesconi 2014, 2016), as well as for legal texts consolidation (Ogawa et al. 2007; Palmirani 2011). In the Semantic Web framework, norms are modeled in terms of ontologies, ontology classes, properties, as well as restrictions on ontology properties able to represent constraints provided by deontic rules (Fig. 2). Such modeling can be used for legal compliance checking and reasoning with logical implications (Francesconi and Governatori 2019; Francesconi 2019).

In this scenario, in my opinion, one of the most relevant research questions is to identify sufficient conditions for implementing legal reasoning within a description logic (DL) framework, like using OWL-DL (as well as DL-Rules and DL-safe Rules) Hitzler et al. (2009). Typical problems that can be addressed are: standard/temporal deontic logic; non-monotonic/defeasible reasoning; argumentation schemes. The identification of such conditions for legal knowledge modeling and legal reasoning within a DL framework is able to guarantee the decidability and the computational tractability of the problem, as well as to rely on an established reasoning algebra. The advantage of this approach is also the possibility to exploit existing description logic reasoners (like Pellet 6, Racer 7, HermiT 8). On the other hand, so far such technologies have hardly scaled for problems of large dimension involving a relevant amount of data. Anyway, this is more a technological problem rather than a theoretical one. In my research, I modeled specific legal reasoning profiles (like Hohfeldian reasoning and legal compliance checking) using description logic (OWL-DL), proving the ability of this approach to deal with defeasible reasoning (Francesconi 2014; Francesconi and Governatori 2019; Francesconi 2019). Other examples do exist in the literature (van de Ven et al. 2008; Gandon et al. 2017), therefore my feeling is that this approach can be generalized, while the quality of knowledge modeling is a key factor for addressing legal reasoning problems within a DL framework.

## Opportunities

**Fig. 3:**
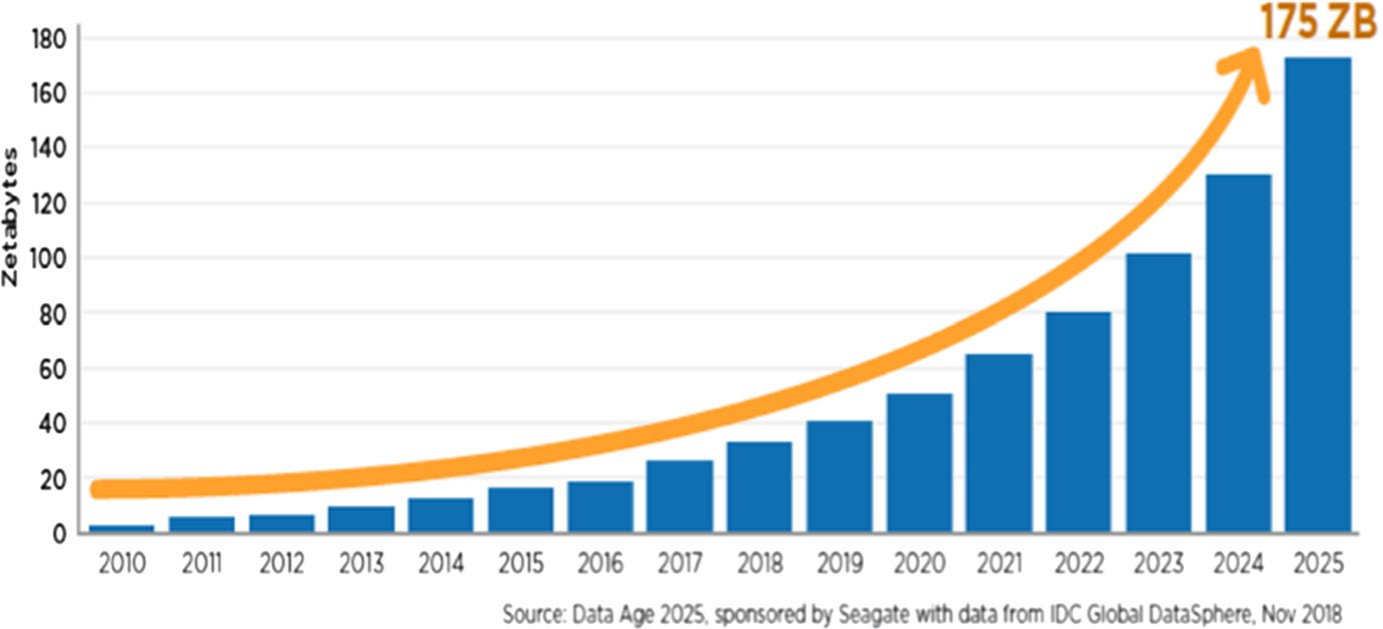
Data growth prediction until 2025

In the current summer of AI and Law, and with the development of the Semantic Web in large scale, we have lots of opportunities to exploit the expected dramatic growth of data (Fig. 3), therefore the aim is to seize the opportunities of the next data wave, combining top-down and bottom-up approaches to AI and Law. On the one hand this basically means using Semantic Web standards for knowledge modeling, on the other hand it means using inference tools for legal reasoning, as well as implementing machine/deep learning facilities for legal knowledge extraction, using smart data for systems evaluation compared to existing baselines.

From the political point of view, European institutions highly support AI development, as pointed out in the recent “White Paper On Artificial Intelligence” of the European Commission (Commission 2020). In such documents the European Commission promotes the creation of excellence and testing centres that can combine European, national and private investments, as well as new public private partnerships in AI, data and robotics, together with the adoption of AI by the Public Sector. The ultimate twofold goal is to create an “ecosystem of excellence”, starting in research and innovation, aimed to create the right incentives to accelerate the adoption of solutions based on AI by small and medium-sized enterprises, as well as a regulatory framework for AI (e.g., data protection, privacy, non-discrimination) (Commission 2020).

The European Commission, in particular, is working on creating the EU open data cloud: one of its cornerstones is Cellar (Francesconi et al. 2015), the main European linked open data repository, centred on EU law, managed by the Publications Office of the European Union. Moreover, recently the European Commission has released a new version of the European Union Open Data Portal 9, while shortly the European Parliament Open Data Portal will be launched as well 10.

These institutional trends go in parallel with the current hype for Legal Tech companies and start-ups, which are rapidly expanding in different sectors: smart contracts, eDiscovery, security and compliance, document management and analysis, business intelligence, case management, workflow tools, legal research, office automation.

This is the scenario envisaged by Richard Susskind, invited speaker at ICAIL 2017. In Susskind (2017) he underlined how “legal institutions and lawyers [...] will change more radically in less than two decades than they have over the past two centuries". The same concept was expressed by Katie Atkinson in her presidential address at ICAIL 2017, when she observed that “plenty of law firms are interested in hearing about what our research can offer”.

## Dreaming in the summer of AI

In the scenario previously described, the link between Knowledge and Intelligence will probably take along the next evolution of AI which therefore, with high probability, will be affected by the evolution of the Web, as well as by the evolution of the machine learning algorithms able to process raw and structured data.

The literature is now unanimous in identifying the next evolution of the Web as the Web 4.0, in which autonomous software agents can interact with each other (machine-to-machine interaction), as well as operate in symbiosis with humans (human-to-machine interaction). For this reason, some authors address the Web 4.0 as *Symbiotic Web*. On the other hand, the environment within which software agents will be able to manage relevant information and interact with humans is sometimes referred to as the *Pragmatic Web*. It represents the specific aspect of Web 4.0 which describes the set of practices and theories according to which people use information acquired on the Web for social interaction, knowledge sharing and participation (Weigand and Arachchig 2010; Weigand and Paschke 2012). In the Web 4.0, it is possible to develop customized software capable of processing structured data. For example, based on the profile and needs of users, a software agent can book a flight at the most appropriate time to get the lowest price, as well as protecting customers with the best alternative option in case of cancellations. Similarly, a customized software agent can select the most interesting news, or make the best economic investment based on the user’s financial risk profile and so on. In such a scenario, when each object can be uniquely identified on the Web (via URI), specific software agents can manage objects’ interaction with humans. A typical example is the one of the self-driving cars that already provide driving support and will, in the near future, have integrated functionalities including owner’s profile, such as his own agenda, so as to better plan routes based on appointments.

Even legal practitioners will be highly influenced by this type of revolution: for example, norms searching and retrieval can be carried out directly by intelligent agents with knowledge of a case in question and of the laws that govern that particular case. As in the Web 3.0 sources of law are represented in a format understandable by machines, in the Web 4.0 nothing prevents us in principle to imagine a scenario in which an intelligent agent assumes the function of Judge who can take the final decision on specific disputes, having knowledge of personal profiles, cases and related regulations. It is a scenario that may seem disturbing, in particular if we consider that the question whether an automatic judge can ever be developed is part of the more general question whether an algorithm, an artificial intelligence, will ever succeed in replacing human legal reasoning. This is a specific case of a broader question whether artificial intelligence will ever replace human intelligence.

The study of Artificial Intelligence, at least in its strong interpretation (namely the attempt to fully replicate the functions of the human brain), had to deal with the limits and opportunities that the human brain model imposes. This model circumscribes the peculiarities of a discipline in which the brain is the cause and the object of research itself, giving the study of AI systems a recursive virtue with surprising consequences.

The first of these consequences is linked to the so-called Hypothesis of Hans Moravec, professor at the Robotic Institute of Carnegie Mellon University, expressed in the essay “Mind Children. The Future of Robot and Human Intelligence” (Moravec 1988). According to Moravec, there exists the *time of human equivalence* $T_0$, that is the moment in which the artificial intelligence will reach the levels of complexity and power of the human mind, such as to make them indistinguishable. It is a scenario anticipated by a large literature of science fiction, a scenario *à la* Philip Dick Dick (1968), *à la* Blade Runner. But paradoxically, if such scenario will ever occur, at time $T_0$ the study of AI would be reduced to the empty set ($\exists T_0$ $\Rightarrow$ AI($T_0$) = $\emptyset$). In the field of Law, this scenario actually opens up the possibility that a machine, on the basis of deductive rules, facts and categories, can reach the levels of complexity of human legal reasoning, until replacing it. But this perspective is not without question marks. For example, does the human judge argue only by deductive categories? Moreover, which role have the emotions in taking decisions? Will a digital judge, emotionally neutral, be fairer than a human judge?

In fact, many scholars believe that the Moravec hypothesis, and the resulting scenario, will never occur, and not for purely technological reasons, as on the contrary the exponential acceleration of the computing power of computers would seem to prefigure, but for reasons of a logical and, so to speak, philosophical nature. These questions not only have value in the field of Law and decision automation, but they are part of a wider range of questions about the very nature of human intelligence. For example, how does one distinguish true from false? How are decisions made? What role do emotions play in decisions? Or is the human mind algorithmic?

Roger Penrose, professor at the Institute of Mathematics at the University of Oxford, gave an enlightening answer to these questions and doubts about the actual occurrence of the Moravec hypothesis, whose conclusions are based on a particular interpretation of the famous “Theorem of Incompleteness” by Kurt Gödel Gödel (1931). This theorem represents an indisputable milestone in the characterization of formal logical systems. In simple terms, Gödel’s theorem states that a coherent (i.e. non-contradictory) system of rules is necessarily incomplete, i.e. there are truths that cannot be proved with the axioms of the system itself. By non-demonstrable statements we mean statements that cannot be derived automatically. Now, a computer is nothing but a set of circuits that reproduce logical (coherent) rules of thought, therefore Gödel’s theorem is valid for it. Therefore, a computer is an incomplete system and, as such, it cannot automatically derive the truth value of every statement.

The key point of Penrose’s reasoning, based on Gödel’s theorem, lies in the observation that human beings, unlike machines, recognize as *true*, things that cannot be derived automatically, that is, they guess the truth of specific statements and create related axioms. Unlike artificial minds, the human mind seems to reconcile coherence and completeness of a rules system: it guarantees the coherence of a system of rules (its non-contradictory nature), while it guesses its completeness (it accepts the axioms by guessing them as *true*). In a nutshell, the human mind guesses its own limits, it is endowed with self-awareness and, as such, it seems to be an exception to Gödel’s theorem! Therefore, according to Penrose, talking about Artificial Intelligence to refer to the automatic replication of human mind is misleading. In fact, there is a risk of assimilating the complexity of human mind, such as conscience, awareness and intuition (in many ways still unknown), to simple logical categories, denying space for creativity and innovation. Penrose, then, summarises his theory in the famous motto “human mind is not algorithmic” (Penrose 1989), meaning that human mind is not a Turing machine. This is like saying that intelligence cannot, by definition, be “artificial”, as intelligence requires awareness, namely the consciousness that machines don’t have.

It is therefore natural to ask whether the studies on AI in the future will have to deal with emotions, intuitions and the automatic production of consciousness. And, similarly, we should ask ourselves if the Web, as a knowledge infrastructure for AI, today emotionally neutral, will have to manage emotions. In this respect, the literature on the future of the Web agrees in believing that the next step of the Web evolution is an infrastructure that makes it possible to distinguish human-machine interaction from machine-machines interaction. Currently, in fact, the information systems on the Web do not perceive user’s emotions and do not react accordingly. Therefore, it seems fascinating to foresee a further evolution of the Web in terms of the Web 5.0 or *Emotional Web* which will include human-machine interaction taking emotions into account. In this context, human beings will be able to communicate their emotions to systems capable of perceiving and processing them, consequently responding to their requests.

In this scenario, the Law would be not an exception: not only rules and facts but also emotions can play a significant role in human-machine interaction. For example, the decision of an automatic judge could also be influenced by the emotional aspects, as it happens today in the interaction between humans. In this scenario, how will a human lawyer be able to persuade an automatic judge?

## Footnotes

1 International Association for AI and Law.

2 International Conference on AI and Law.

3 through the Institute of Legal Informatics which has changed its name over the years (IDG, ITTIG, IGSG).

4 Logic, Informatics and Law.

5 first IAAIL president.

6 https://github.com/stardog-union/pellet.

7 https://www.ifis.uni-luebeck.de/ moeller/racer/.

8 https://github.com/phillord/hermit-reasoner.

9 https://data.europa.eu.

10 it will be available at https://data.europarl.europa.eu.

## References

[CR1] Agnoloni T, Bacci L, Francesconi E, Spinosa P, Tiscornia D, Montemagni S, Venturi G (2007) Building an ontological support for multilingual legislative drafting In: Proceedings of the Jurix Conference, pp 9–18

[CR2] Aleven V, Ashley K (1997) Evaluating a learning environment for case-based argumentation skills In: Proceedings of the Sixth International Conference on Artificial Intelligence and Law pp 170–179 ACM Press

[CR3] Antoniou G, Dimaresis N, Governatori G (2008) A system for modal and deontic defeasible reasoning In: Proceedings of the 2008 ACM Symposium on Applied Computing pp 2261–2265 ACM 10.1145/1363686.1364226

[CR5] Ashley K (1991) Reasoning with cases and hypotheticals in hypo Int J Man Mach Stud 753–796

[CR6] Ashley K, Sartor G, Casanovas P, Biasiotti M, Fernández-Barrera M (2011). the case-based reasoning approach ontologies for analogical legal argument. Approaches to legal: ontologies law governance and technology series.

[CR7] Atkinson K, Baroni P, Giacomin M, Hunter A, Prakken H, Reed C, Simari G, Thimm M, Villata S (2017). Toward artificial argumentation. AI Mag.

[CR8] Atkinson K, Bench-Capon TJM, Bollegala D (2020) Explanation in ai and law: past, present and future. Artif Intell 289:103387. 10.1016/j.artint.2020.103387

[CR4] Barredo Arrieta A, Díaz-Rodríguez N, Del Ser J, Bennetot A, Tabik S, Barbado A, Garcia S, Gil-Lopez S, Molina D, Benjamins R, Chatila R, Herrera F (2020) Explainable Artificial Intelligence (XAI): concepts, taxonomies, opportunities and challenges toward responsible AI. Inf Fusion 58:82–115. 10.1016/j.inffus.2019.12.012

[CR9] Bench-Capon T (1989) Deep models, normative reasoning and legal expert systems In: Proceedings of the second international conference on artificial intelligence and law pp 37–45 ACM Press, New York

[CR10] Bench-Capon T (1993) Neural networks and open texture In: Proceedings of the fourth international conference on AI and Law, pp 292–297 ACM Press, New York

[CR11] Bench-Capon T, Gordon T (2009) Ismorphism and argumentation In: Proceedings of the twelfth international conference on artificial intelligence and law, pp 11–20 ACM Press, New York

[CR12] Bex F (2015) An integrated theory of causal stories and evidential arguments In: ACM (ed) Proceedings of the 15th International Conference on Artificial Intelligence and Law, pp 13–22

[CR13] Bhattacharya P, Poddar S, Rudra K, Ghosh K, Ghosh S (2021) Incorporating domain knowledge for extractive summarization of legal case documents Proceedings of the 18th International Conference on Artificial Intelligence and Law

[CR14] Biagioli C (2009) Modelli Funzionali delle Leggi. Verso testi legislativi autoesplicativi, Legal Information and Communications Technologies Series, vol. 6 European Press Academic Publishing, Florence

[CR15] Biagioli C, Cappelli A, Francesconi E, Turchi F (2007) Law making environment: perspectives In: Proceedings of the V Legislative XML Workshop, pp 267–281 European Press Academic Publishing

[CR16] Biagioli C, Francesconi E, Passerini A, Montemagni S, Soria C (2005) Automatic semantics extraction in law documents In: International Conference on Artificial Intelligence and Law, pp 133–139

[CR17] Biagioli C, Francesconi E, Spinosa P, Taddei M (2005) A legal drafting environment based on formal and semantic xml standards In: International Conference on Artificial Intelligence and Law, pp 244–245

[CR18] Bochereau L, Bourcier D, Bourgine P (1991) Extracting legal knowledge by means of a multilayer neural network application to municipal jurisprudence In: Proceedings of the 3rd International Conference on Artificial intelligence and Law

[CR19] Boer A, Hoekstra R, de Maat E, Hupkes E, Vitali F, Palmirani M (2010) Cen workshop agreement ‘open xml interchange format for legal and legislative resources’ Tech Rep CWA 15710:2010 E, CEN European Committee for Standardization

[CR20] Branting LK (1991). Building explanations from rules and structured cases. Int J Man Mach Stud.

[CR21] Casanovas P, Palmirani M, Peroni S, Engers TV, Vitali F (2016). Semantic web for the legal domain: the next step. Semant Web J.

[CR22] ceur.org (ed): Proceedings of the Workshop on Automated Semantic Analysis of Information in Legal Texts (2015-2021)

[CR23] Commission European (2020) On artificial intelligence - a european approach to excellence and trust Tech rep, European Commission

[CR24] Conrad JG (2010) E-discovery revisited: the need for artificial intelligence beyond information retrieval Artif Intell Law (Special Issue on "E-Discovery") **18**(4), 321–345

[CR25] Conrad JG, Zeleznikow J (2015) The role of evaluation in ai and law: An examination of its different forms in the ai and law journal In: Proceedings of the 15th International Conference on Artificial Intelligence and Law, pp 181–186 San Diego, CA, ACM Press

[CR26] de Maat E, Krabben K, Winkels R (2010) Machine learning versus knowledge based classification of legal texts In: Proceedings of the Jurix Conference: legal Knowledge and Information Systems, pp 87–96 IOS Press, The Netherlands

[CR27] Devlin J, Chang M.W., Lee K, Toutanova K (2019) BERT: pre-training of deep bidirectional transformers for language understanding In: Burstein J, Doran C, Solorio T (eds) Proceedings of the 2019 Conference of the North American Chapter of the Association for Computational Linguistics: human Language Technologies, NAACL-HLT 2019, vol. 1, pp 4171–4186 Minneapolis, MN, USA,

[CR28] Dick PK (1996) Do androids dream of electric sheep? New York : Ballantine Books (1996 ($$\copyright$$ 1968))

[CR29] Francesconi E (2016) Semantic model for legal resources: annotation and reasoning over normative provisions Semantic Web journal: Special Issue on Semantic Web for the legal domain 7(3):255–265

[CR30] Francesconi E (2019) Reasoning with deontic notions in a decidable framework In: Peruginelli G, Faro S (eds) Knowledge of the law in the big data age, Frontiers in Artificial Intelligence and Applications, vol 317 IOS Press, pp 63–77

[CR31] Francesconi E (2014). A description logic framework for advanced accessing and reasoning over normative provisions. Int J Artif Intell Law.

[CR32] Francesconi E, Passerini A (2007). Automatic classification of provisions in legislative texts. Int J Artif Intell Law.

[CR33] Francesconi E, Governatori G (2019) Legal compliance in a linked open data framework. In: Legal Knowledge and Information Systems, pp 175–180 IOS Press

[CR34] Francesconi E, Küster M, Gratz P, Thelen S (2015) The ontology-based approach of the Publications Office of the EU for document accessibility and open data services In: A. Kö, E. Francesconi (eds.) Proceedings of the 4rd International Conference on Electronic Government and the Information Systems Perspective, pp 29–39. Valencia, Spain

[CR35] Gandon F, Governatori G, Villata S (2017) Normative requirements as linked data In: Wyner A, Casini G (eds) Legal Knowledge and Information Systems - Proceeding of the JURIX Conference, vol 302 IOS Press, pp 1–10

[CR36] Gardner A (1987). An artificial intelligence approach to legal reasoning.

[CR37] Gödel K (1931). über formal unentscheidbare sätze der principia mathematica und verwandter systeme. Monatshefte für Mathematik und Physik.

[CR38] Gordon T (1987) Oblog-2: a hybrid knowledge representation system for defeasible reasoning In: Proceedings of the First International Conference on Artificial Intelligence and Law

[CR39] Gordon T (1988) The importance of nonmonotonicity for legal reasoning In: Fiedler H, Haft F, Traunmüller R (eds) expert systems in law: impacts on legal theory and computer law Attempto-Verlag, Tübingen, pp 111–126

[CR40] Guastini R (2010). Le Fonti del Diritto.

[CR41] Hitzler P, Krötzsch M, Rudolph S (2009) Foundations of the semantic web technologies. Chapman & Hall/CRC

[CR42] Lauristen M (2015). On balance. Artif Intell Law.

[CR43] Lenci A, Montemagni S, Pirrelli V, Venturi G (2007) Nlp-based ontology learning from legal texts a case study In: Casanovas P, Biasiotti MA, Francesconi E and Sagri MT (Eds), Proceedings of LOAIT 07 - II Workshop on Legal Ontologies and Artificial Intelligence Techniques, pp 113–129

[CR44] Loevinger L (1949) Jurimetrics: The next step forward Minnesota Law Rev 33(1796)

[CR45] Marmor A (2014). The language of law.

[CR46] McCarty L (1984) Intelligent legal information systems: problems and prospects In: Campbell C (ed) Data processing and the Law Sweet and Maxwell, London, pp 125–151

[CR47] McCarty LT (1990). Artificial intelligence and law: how to get there from here. Ratio Juris.

[CR48] Mikolov T, Sutskever I, Chen K, Corrado G, Dean J (2013) Distributed representations of words and phrases and their compositionality In: Proceedings of the 26th International Conference on Neural Information Processing Systems, vol 2, pp. 3111–3119

[CR49] Minsky M, Papert S (1969). Perceptrons.

[CR50] Mochales Palau R, Moens MF (2009) Argumentation mining: the detection, classification and structure of arguments in text In: Proceedings of the Twelfth International Conference on Artificial Intelligence and Law, pp 98–109 ACM

[CR51] Moravec H (1988). Mind children: the future of robot and human intelligence.

[CR52] Ogawa Y, Inagaki S, Toyama K (2007) Automatic consolidation of Japanese statutes based on formalization of amendment sentences In: JSAI ’07, pp 363–376

[CR53] Palmirani M (2011) Legislative change management with akoma-ntoso In: Sartor G, Palmirani M, Francesconi E, Biasiotti MA (eds) Legislative XML for the Semantic Web: principles, models, standards for document management, law, governance and technology series, vol. 4, chap 7, pp 101–130 Springer, Netherlands

[CR54] Penrose R (1989). The emperor’s new mind: concerning computers, minds, and the laws of physics.

[CR55] Peters W, Wyner A (2016) Legal text interpretation: Identifying hohfeldian relations from text In: Proceedings of the 10th International Conference on Language Resources and Evaluation, pp 379–384 European Language Resources Association (ELRA)

[CR56] Prakken H, Sartor G (1997). Argument-based extended logic programming with defeasible priorities. J Appl Non Classic Logics.

[CR57] Prakken H, Sartor G (1996) A dialectical model of assessing conflicting arguments in legal reasoning Logical models of legal argumentation Springer, Dordrecht, pp 175–211

[CR58] Raz J (1980). The concept of a legal system.

[CR59] Rich E, Knight K (1991) Artificial Intelligence (2nd edn). Tata McGraw Hill

[CR60] Rissland E, Skalak D (1991). Cabaret: statutory interpretation on a hybrid architecture. Int J Man Mach Stud.

[CR61] Rissland E, Ashley K (1987) A case-based system for trade secrets law In: Proceedings of the First International Conference on Artificial Intelligence and Law, pp 60–66 ACM Press

[CR62] Rissland E, Skalak D (1989) Combining case-based and rule-based reasoning: a heuristic approach In: Proceedings of 11th International Joint Conference on Artificial Intelligence, pp 524–530 Morgan Kaufmann, CA

[CR63] Rosenblatt F (1958). The perceptron: a probabilistic model for information storage and organization in the brain. Psychol Rev.

[CR64] Sartor G (2016) L’informatica giuridica e le tecnologie dell’informazione Giappichelli

[CR65] Sartor G (2006). Fundamental legal concepts: a formal and teleological characterisation. Artif Intell Law.

[CR66] Savelka J, Westermann H, Benyekhlef K, Alexander CS, Grant JC, Amariles DR, Hamdani RE, Meeùs S, Troussel A, Araszkiewicz M, Ashley KD, Ashley A, Branting K, Falduti M, Grabmair M, Harašta J, Novotná T, Tippett E, Johnson S (2021) Lex rosetta: transfer of predictive models across languages, jurisdictions, and legal domains In: ACM (ed.) Proceedings of the Eighteenth International Conference on Artificial Intelligence and Law, pp 129–138

[CR67] Susskind R (2017). Tomorrow’s lawyers.

[CR68] van de Ven S, Breuker J, Hoekstra R, Wortel L (2008) Automated legal assessment in owl 2 In: I Press (ed.) JURIX 2008: the Twenty-First annual conference on legal knowledge and information systems, pp 170–175

[CR69] Verheij B (2020). Artificial intelligence as law. Artif Intell Law.

[CR70] Waltl B, Muthur J, Glaser I, Bonczek G, Scepankova E, Matthes F (2017) Classifying legal norms with active machine learning In: Wyner A, Casini G (eds) Legal Knowledge and Information Systems - Proceeding of the JURIX Conference. IOS Press, pp 11–20

[CR71] Walton D (2006). Fundamentals of critical argumentation.

[CR72] Weigand H, Arachchig JJ (2010) Value network analysis for the pragmatic web: a case of logistic innovation In: I-Semantics ACM

[CR73] Weigand H, Paschke A (2012) The pragmatic web: Putting rules in context In: AGe A Bikakis (ed) Rules on the web: research and applications (RuleML 2012), Lecture Notes in Computer Science, vol 7438 Springer, Berlin, Heidelberg

[CR74] Winkels R, J d R (2011) Survival of the fittest: Network analysis of dutch supreme court cases International Workshop on AI Approaches to the Complexity of Legal Systems Springer, Berlin, Heidelberg, pp 106–115

